# Food Consumption Patterns and Sedentary Behaviors Among the University Students: A Cross‐Sectional Study

**DOI:** 10.1002/hsr2.70259

**Published:** 2024-12-17

**Authors:** Mst. Mahfuza Akter, Md. Jubayer Hossain

**Affiliations:** ^1^ Population Health Studies Division, Centre for Health Innovation, Research, Action and Learning—Bangladesh (CHIRAL Bangladesh) Dhaka Bangladesh; ^2^ Department of Community Nutrition Bangladesh University of Health Sciences Dhaka Bangladesh

**Keywords:** Bangladesh, food consumption, sedentary behaviors, student lifestyle, university students

## Abstract

**Background:**

University is a critical period regarding unhealthy changes in eating behaviors in students. University students often face significant changes in their eating habits and physical activity levels, which can impact their overall health.

**Aims:**

To investigate the eating habits and sedentary behavior of university students in Dhaka.

**Methods:**

This research was based on a cross‐sectional study. The snowball sampling technique was applied to survey university students in Dhaka city. Structured questionnaires were used for data collection through an online survey. Data collection was done from November 2022 to April 2023. Descriptive statistics, including frequency, percentage, mean, and standard deviation (SD), were computed for the variables of interest. Chi‐squared test was performed to estimate the association between the participants' eating habits, sedentary behaviors, and BMI and eating habits. In all analyses, a *p*‐value of < 0.05 was considered statistically significant.

**Results:**

A total of 444 students participated in this study; 44% of them consumed breakfast irregularly. Only 25% of students favored vegetables. About 55% of students preferred junk food. Furthermore, only 19% of students consumed fruits daily. This study also found that a substantial proportion of students used the internet and mobile phones for long periods and didn't practice physical activity. According to the Chi‐squared test, dinner habits (*p* < 0.001), smoking status (*p* < 0.001), alcohol consumption (*p* = 0.014), watching television (*p* = 0.023), and practicing physical activity (*p* = 0.023) had a significant association with eating habits and sedentary behaviors amongst the participants.

**Conclusions:**

Overall, most participants in this study exhibited commendable dietary patterns, except for meal frequency, fruit intake, consumption of junk food, and fried food. However, their physical activity levels were notably inadequate, largely characterized by a sedentary lifestyle involving substantial internet and mobile phone usage.

## 1 Introduction

Bangladesh, like other Southeast Asian countries, has experienced an epidemiological transition, resulting in marked changes in the population's food consumption patterns and lifestyle behaviors [1, 2]. Traditional eating patterns have been replaced with Westernized food, which includes low dietary fiber, vegetables, and fruit consumption, as well as high‐fat, sugar, and salt meals. In addition, university students' fast food intake is increasing [3], which typically has a high calorie, fat, and salt and has become an essential item in Bangladeshi meals [4]. Several studies in Bangladesh have indicated that adolescents and young people have unhealthy eating patterns, such as skipping breakfast, consuming fewer fruits, vegetables, milk, and fish, and consuming more sugar‐sweetened beverages, fast food, and sweets [3, 4, 5, 6].

Furthermore, it has been observed that university students spend considerable time sedentary. According to self‐reports from 32 studies, university students spend an average of 7.29 h per day in sedentary activities, with some studies revealing significantly greater levels when measured with devices such as accelerometers [7, 8]. Physical inactivity is responsible for approximately 3.2 million deaths per year [9]. This global pandemic of physical inactivity is present in 16.6%–34.5% of adults [10]. It should be highlighted that the rate of inactivity among Bangladeshi adults is overall 34.5%, in urban 37.7%, and 31.6% in rural areas, respectively [11]. A higher proportion of university students are sedentary globally because they spend much time online on social media, watching television, accessing the internet, playing computer games, shopping online, or studying [12, 13, 14]. This high level of inactivity can lead to various health issues, including an increased risk of chronic disease and mental health problems [8].

Over the past two decades, the incidence of obesity in emerging nations has tripled because of urbanization, changes in eating habits, and the adoption of a more sedentary lifestyle [15, 16]. In 2022, worldwide, 2.5 billion adults were overweight, including 890 million who were living with obesity, while 390 million were underweight [17]. According to the Global Nutrition Report (2019), in the Southeast, about 27.5% of males and females, 32.1%, were overweight, and 6.4% of males, female 10.2%, were obese [18]. In a recent study in Bangladesh, among university students, the prevalence rates overweight at 9.7% and obese at 3.2% [19]. This condition is associated with a higher incidence of hypertension, diabetes mellitus, arthritis, gout, and gallbladder problems [20, 21].

Studies have shown that sedentary behavior, such as prolonged sitting and low physical activity, is often associated with poor eating habits [22, 23]. Students who spend more time in sedentary activities tend to consume higher calorie, low nutrient foods like fast food, sugary snacks, beverages, and skip meals, particularly breakfast, and have an irregular eating pattern [23]. In contrast, a healthy diet and physical activity are vital in human life; it has many positive benefits, such as preventing heart disease, cancer, diabetes, hypertension, dyslipidemia, peptic ulcers, and many other diseases caused by unhealthy food habits or physical inactivity [24, 25, 26, 27].

Universities may represent a setting where dietary behaviors are open to change, and large groups of young adults can be reached, making them an appropriate target for health promotion efforts. A dietary pattern has been used widely in various Bangladeshi population groups but has not been employed to characterize university students' dietary patterns and sedentary behavior. Therefore, this study aimed to investigate university students' eating habits and sedentary behavior in Dhaka, Bangladesh. These findings could be useful to help understand the distribution of eating habits and sedentary behaviors among university students and to consider possible preventive measures.

## 2 Materials and Methods

### 2.1 Study Design

This cross‐sectional study was conducted from November 2022 to April 2023 among university students in Dhaka, Bangladesh.

### 2.2 Setting and Participants

Using snowball sampling, 444 university students were included in this study. The following eligibility criteria were considered when recruiting the participants: (i) being Bangladeshi by birth; (ii) age group (18–35) years; (iii) current university student of Dhaka city. The formula is *n* = *z* ^2^ *pq*/*d* ^2^ (where 95% level of confidence *z* = 1.96, *p* = the expected proportion of sedentary behaviors In Bangladesh, 20.9% [27]; *q* = 1 − *p*; and 5% margin of error *d* = 0.05) were used to determine the sample. Considering the nonresponse rate of 5%, the total number of samples was 254 + 12 ≈ 266. To increase the external validity and generalizability of the study, we included more participants than the calculated sample size [28]. Eleven students dropped out of 455 due to their non‐cooperation, ignorance of information, forgetting to submit Google forms on time, and less interest. Finally, 444 participants were included in this study.

### 2.3 Data Collection Instruments

The researcher designed a three‐part, self‐administered questionnaire. Part 1 of the questionnaire sought sociodemographic and anthropometric information, such as height and weight. Part 2 assessed dietary behavior, eating, drinking, and smoking habits. The questionnaire was adopted from a previously published study [5, 29, 30] and was standardized for university students. Part 3 focused on measuring sedentary behaviors. The questionnaire was also adopted from a previously published study that checked the reliability [31]. The result showed a Pearson correlation coefficient of 0.87, indicating good reliability [31].

### 2.4 Ethical Consideration

The ethical research committee approved this study's protocol at the Center for Health Innovation, Research, Action, and Learning, Bangladesh (CHIRAL Bangladesh) (reference number: CHIBAN02DEC2022‐001). The consent obtained from participants was in written form (online). This approach was chosen to ensure a verifiable and formal agreement from the participants, aligning with ethical research standards. Participants received a detailed consent form outlining the study's purpose, procedures, potential risks, and confidentiality agreements. The online survey began with the respondent's informed consent and eligibility verification. It was made clear that study participation was voluntary.

### 2.5 Data Collection Procedure and Measurements

Google Forms was used to create the survey questionnaire. The survey link was shared with potential participants through appropriate channels, such as email invitations and on social media (Facebook, Messenger, and WhatsApp), requesting participation. Recipients were asked to share the link with their connections, and only those currently studying at the university in Dhaka city were asked to do so. The data collection procedure followed the ethical guidelines of the University of Helsinki for research involving human subjects.

### 2.6 Body Mass Index (BMI)

BMI was calculated from self‐perceived height and weight; BMI = (kg/m^2^). According to the World Health Organization (WHO) guidelines for the Asian population, BMI was classified into four groups: underweight (BMI < 18.5 kg/m^2^), normal weight (BMI = 18.5–24.9 kg/m^2^), and overweight (BMI = 25.0–29.9 kg/m^2^), and obese (BMI > 30 kg/m^2^) [32].

### 2.7 Statistical Analysis

Data analysis was conducted using R programming (version 4.2.0). The (Mean ± SD) was to present continuous variables. Count and percentage were used to present categorical data. Chi‐squared test was performed to estimate the association between the participants' eating habits, sedentary behaviors, and BMI and eating habits. In all analyses, a *p*‐value of < 0.05 was considered statistically significant.

## 3 Results

### 3.1 Demographic Characteristics of the Participants

The total number of participants with a mean age of 21.73 (SD: 2.21) years and a BMI mean of 22.68 (SD: 4.16) kg/m^2^ were included in this study. More than half (63%) of the participants were females. The majority (91%) of the participants were unmarried. More than half (59%) of the participants majored in science, 11% in business, 10% in art and humanities, and social sciences 20%. The results showed 24% lower Socioeconomic (< 15,000 BDT) status, 45% middle Socioeconomic (15,000–30,000 BDT), and 31% upper socioeconomic (> 30,000 BDT), respectively (Table 1).

**Table 1:** Demonstrates the demographic characteristics of the participants (*N* = 444).

Variables	*n* (%)
Age (years) (M ± SD)	21.73 ± 2.21
Height (m) (M ± SD)	1.62 ± 0.10
Weight (kg) (M ± SD)	59.69 ± 12.65
BMI (kg/m^2^) (M ± SD)	22.68 ± 4.16
Gender	
Male	165 (37)
Female	279 (63)
Marital status	
Married^a^	40 (9)
Unmarried	404 (91)
Field of study	
Science	264 (59)
Business	51 (11)
Arts and humanities	43 (10)
Social Science	86 (20)
Socioeconomic status	
Lower socioeconomic (< 15,000 BDT)	109 (24)
Middle socioeconomic (15,000–30,000 BDT)	198 (45)
Upper socioeconomic (> 30,000 BDT)	137 (31)

^a^ Married included (Married, Divorced, and Widow). M = mean and SD = standard deviation. Descriptive statistics were done.

### 3.2 BMI Status of the Participants

**Figure 1:**
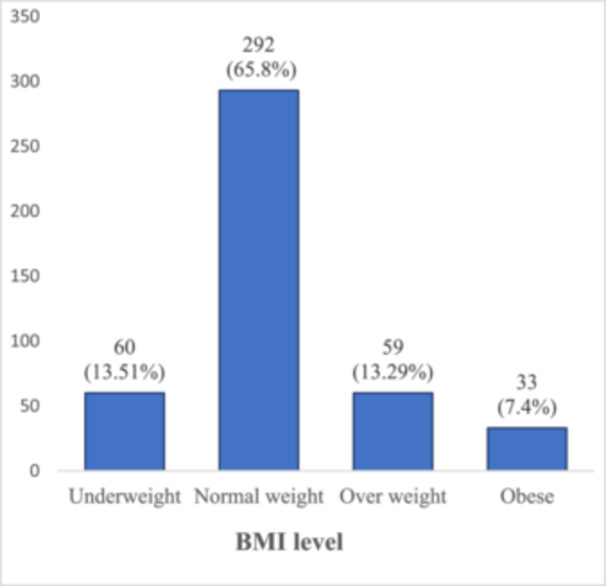
Distribution of BMI percentage among the participants (*N* = 444).

Figure 1 shows that the majority (65.8%) of participants were of normal weight; the proportions of underweight, overweight, and obese were 13.51%, 13.29%, and 7.4%, respectively.

### 3.3 Eating Habits of the Participants (Table 2)

**Table 2:** Eating habits among the participants based on gender (*N* = 444).

Variables	Male	Female	Total	*p*‐value
	*n* (%)	*n* (%)	*n* (%)	
Do you take morning breakfast?				0.6
Always	48 (29)	95 (34)	143 (32)	
Regularly	41 (25)	66 (24)	107 (24)	
Irregular	76 (46)	118 (42)	194 (44)	
Do you take lunch?				0.4
Always	74 (45)	111 (40)	185 (42)	
Regularly	63 (38)	106 (38)	169 (38)	
Irregular	28 (17)	62 (22)	90 (20)	
Do you take dinner?				< 0.001*
Always	91 (55)	96 (34)	187 (42)	
Regularly	58 (35)	98 (35)	156 (35)	
Irregular	16 (10)	85 (31)	101 (23)	
What type of food do you prefer most?				0.3
Rice meat	98 (59)	154 (55)	252 (57)	
Vegetables	44 (27)	69 (25)	113 (25)	
Other	23 (14)	56 (20)	79 (18)	
How often do you eat your favorite food daily?				0.011*
One to two times	115 (70)	227 (81)	342 (77)	
Three to four times	43 (26)	41 (15)	84 (19)	
More than four times	7 (4)	11 (4)	18 (4)	
Do you prefer junk food?				0.5
Yes	88 (53)	158 (57)	246 (55)	
No	77 (47)	121 (43)	198 (45)	
How often do you eat junk food?				0.3
Daily	25 (15)	28 (10)	53 (12)	
Once to twice per week	73 (45)	115 (41)	188 (42)	
Three or four times per week	22 (13)	47 (17)	69 (16)	
Rarely	45 (27)	89 (32)	134 (30)	
How often do you eat fruits?				0.7
Daily	36 (22)	49 (18)	85 (19)	
Once to twice per week	59 (36)	102 (36)	161 (36)	
Three or four times per week	31 (19)	61 (22)	92 (21)	
Rarely	39 (23)	67 (24)	106 (24)	
How often do you eat fried food?				0.10
Daily	43 (26)	54 (19)	97 (21)	
Once to twice per week	60 (36)	116 (42)	176 (40)	
Three or four times per week	34 (21)	44 (16)	78 (18)	
Rarely	28 (17)	65 (23)	93 (21)	
How often do you eat outside?				0.2
Daily	38 (23)	46 (16)	84 (19)	
Once to twice per week	51 (31)	94 (34)	145 (33)	
Three or four times per week	31 (19)	45 (16)	76 (17)	
Rarely	45 (27)	94 (34)	139 (31)	
Mention your preferred cooking method				0.2
Boiled	17 (10)	19 (7)	36 (8)	
Grilled	24 (15)	26 (9)	50 (11)	
Traditional	108 (65)	207 (74)	315 (71)	
Other	16 (10)	27 (10)	43 (10)	
Please state your smoking history				< 0.001*
Current smoker	36 (22)	22 (8)	58 (13)	
Ex‐smoker	16 (10)	13 (5)	29 (7)	
Never smoke	113 (68)	244 (87)	357 (80)	
Do you drink alcohol?				0.014*
Yes	40 (9)	18 (6)	22 (13)	
No	404 (91)	261 (94)	143 (87)	

*Note:* Chi‐squared test was done.

* *p*‐value < 0.05 was considered significant.

Among the participants, approximately 44% reported irregular meal consumption at breakfast, while 20% were irregular at lunch and 23% at dinner, indicating varying patterns of meal irregularity. A significant association was found between dinner habits and gender (*p* < 0.001). Additionally, 57% of participants favored rice and meat as their favorite food, with a significant association between food preference frequency and gender (*p* = 0.011). Over half (55%) of participants preferred junk food, while only 19% consumed fruits daily. About 40% of participants consumed fried food once to twice per week, but a majority (71%) of the participants preferred traditional cooking methods. Smoking behaviors also had a significant association by gender (*p* < 0.001), with 13% of participants being current smokers. Furthermore, alcohol consumption (*p* = 0.014) was a significant association between the gender.

### 3.4 Association Between BMI and Eating Habits of the Participants

The association between BMI and participants' eating habits demonstrated that BMI was significantly associated with lunchtime (*p* = 0.043). However, none of the other dietary pattern parameters was (*p* > 0.05) associated with the participants' BMI (Table 3).

**Table 3:** Demonstrate the association between BMI categories and eating habits of the participants (*N* = 444).

Variables	Under weight	Normal weight	Over weight	Obese	Total	*p*‐value
	*n* (%)	*n* (%)	*n* (%)	*n* (%)	*n* (%)	
Do you take morning breakfast?						0.062
Always	24 (40)	94 (32)	16 (27)	9 (27)	143 (32)	
Regularly	12 (20)	78 (27)	7 (12)	10 (31)	107 (24)	
Irregular	24 (40)	120 (41)	36 (61)	14 (42)	194 (44)	
Do you take lunch?						0.043*
Always	26 (43)	120 (41)	21 (35)	18 (55)	185 (42)	
Regularly	20 (33)	118 (40)	18 (31)	13 (39)	169 (38)	
Irregular	14 (24)	54 (19)	20 (34)	2 (6)	90 (20)	
Do you take dinner?						0.7
Always	27 (45)	123 (42)	22 (37)	15 (45)	187 (42)	
Regularly	22 (37)	104 (36)	18 (31)	12 (37)	156 (35)	
Irregular	11 (18)	65 (22)	19 (32)	6 (18)	101 (23)	
What type of food do you prefer most?						0.3
Rice Meat	34 (57)	164 (56)	31 (52)	23 (70)	252 (57)	
Vegetables	14 (23)	76 (26)	14 (24)	9 (27)	113 (25)	
Other	12 (20)	52 (18)	14 (24)	1 (3)	79 (18)	
How often do you eat your favorite food daily?						0.15
One to two times	52 (87)	226 (77)	39 (66)	25 (76)	342 (77)	
Three to four times	7 (12)	52 (18)	17 (29)	8 (24)	84 (19)	
More than four times	1 (1)	14 (5)	3 (5)	0 (0)	18 (4)	
Do you prefer junk food?						0.7
Yes	37 (62)	157 (54)	34 (58)	18 (55)	246 (55)	
No	23 (38)	135 (46)	25 (42)	15 (45)	198 (45)	
How often do you eat junk food?						0.9
Daily	6 (10)	36 (12)	7 (12)	4 (12)	53 (12)	
Once to twice per week	26 (43)	119 (41)	27 (46)	16 (48)	188 (42)	
Three or four times per week	12 (20)	43 (15)	10 (17)	4 (13)	69 (16)	
Rarely	16 (27)	94 (32)	15 (25)	9 (27)	134 (30)	
How often do you eat fruits?						0.5
Daily	12 (20)	51 (17)	14 (24)	8 (24)	85 (19)	
Once to twice per week	20 (33)	108 (37)	21 (35)	12 (37)	161 (36)	
Three or four times per week	12 (20)	66 (23)	6 (10)	8 (24)	92 (21)	
Rarely	16 (27)	67 (23)	18 (31)	5 (15)	106 (24)	
How often do you eat fried food?						0.7
Daily	11 (18)	61 (21)	15 (25)	10 (30)	97 (22)	
Once to twice per week	24 (40)	114 (39)	25 (43)	13 (40)	176 (40)	
Three or four times per week	14 (24)	54 (18)	7 (12)	3 (9)	78 (17)	
Rarely	11 (18)	63 (22)	12 (20)	7 (21)	93 (21)	
How often do you eat outside?						0.3
Daily	13 (22)	50 (17)	12 (21)	9 (27)	84 (19)	
Once to twice per week	17 (28)	90 (31)	22 (37)	16 (49)	145 (33)	
Three or four times per week	12 (20)	53 (18)	9 (15)	2 (6)	76 (17)	
Rarely	18 (30)	99 (34)	16 (27)	6 (18)	139 (31)	
Mention your preferred cooking method						0.5
Boiled	4 (6)	25 (9)	3 (5)	4 (12)	36 (8)	
Grilled	7 (12)	27 (9)	12 (20)	4 (12)	50 (11)	
Traditional	43 (72)	208 (71)	41 (70)	23 (70)	315 (71)	
Other	6 (10)	32 (11)	3 (5)	2 (6)	43 (10)	
Please state your smoking history						0.2
Current smoker	9 (15)	31 (11)	11 (19)	7 (21)	58 (13)	
Ex‐smoker	2 (3)	20 (6)	4 (6)	3 (9)	29 (7)	
Never smoker	49 (82)	241 (83)	44 (75)	23 (70)	357 (80)	
Do you drink alcohol?						0.2
Yes	5 (8)	22 (8)	7 (12)	6 (18)	40 (9)	
No	55 (92)	270 (92)	52 (88)	27 (82)	404 (91)	

*Note:* Chi‐squared test was done.

* *p*‐value < 0.05 was considered significant.

### 3.5 Sedentary Behaviors of the Participants

Sedentary behaviors among the participants by gender are shown (Table 4). Approximately 16% of participants reported watching television for more than 2 h per day, with a statistically significant association between the number of hours spent watching television per day and gender (*p*  =  0.023). Additionally, a higher proportion of participants used internet and mobile phones frequently. In contrast, only 19% of participants engaged in daily physical activity, with a significant association between physical activity and gender (*p* = 0.023). Furthermore, 51% of the participants slept less than 7 h daily.

**Table 4:** Sedentary behaviors among participants according to gender (*N* = 444).

Variables	Male	Female	Total	*p*‐value
	*n* (%)	*n* (%)	*n* (%)	
Hours of watching television/day				0.023*
< 2	131 (79)	244 (87)	375 (84)	
2+	34 (21)	35 (13)	69 (16)	
Hours of using internet/day				0.8
< 3	25 (15)	45 (16)	70 (16)	
3+	140 (85)	234 (84)	374 (84)	
Hours of using mobile phone/day				0.9
< 2	17 (10)	30 (11)	47 (11)	
2+	148 (90)	249 (89)	397 (89)	
Frequency of practicing physical activity				0.023*
Daily	43 (26)	43 (15)	86 (19)	
Infrequently	49 (30)	95 (34)	144 (33)	
No practice	73 (44)	141 (51)	214 (48)	
Hours of sleeping/day				0.2
< 7	90 (55)	135 (48)	225 (51)	
7+	75 (45)	144 (52)	219 (49)	

*Note:* Chi‐squared test was done.

* *p*‐value < 0.05 was considered significant.

## 4 Discussion

In this cross‐sectional study was to assess the eating habits and sedentary behaviors of the participants. The study found that many participants irregularly consumed meals, particularly breakfast, large consumption of fast food, and low consumption of fruits and vegetables. It has also been observed that most of the student's physical activity levels were notably inadequate, largely characterized by a sedentary lifestyle involving substantial usage of the internet and mobile phones. These unhealthy eating habits and sedentary behaviors can lead to undernourishment or over‐nourishment, increasing susceptibility to avoidable diseases.

This study found that the proportion of overweight and obesity was 13.29% and 7.4%. These findings align with studies conducted in the northern part of Bangladesh, Lebanon, and Malaysia [33, 34, 35]. These findings reflected that participants may consume excessive fast food or be physically inactive.

University students typically do not have healthy eating habits. In this study, most participants skipped breakfast. Similar findings reported in the previous study [5]. Conversely, Malaysian research revealed that 43.9% of Malay medical students consume breakfast daily [35]. Breakfast is crucial for body health and well‐being. In this study, participants may find it challenging to eat breakfast as they are always in a hurry to go to their classes. Some may deliberately skip breakfast because they are conscious of their body weight and appearance.

The present study revealed that about 42% of the respondents always took lunch. This study's findings are significantly lower than the Chinese, Malaysia, and Lebanon study findings [34, 35, 36]. These disparities in findings could plausibly cause participants to skip lunch because they presented at a time on the university campus in Bangladesh due to a packed class schedule, and many university cafeterias or nearby cafeterias may not always offer nutritious lunch options or healthy foods.

This study found that only 25% of students preferred vegetables. In contrast, at Patuakhali Science and Technology University, Patuakhali, Bangladesh, respondents preferred vegetables 69% [5], in China 47.9% [36], and in Bahrain 26.2% [31]. Perhaps university students skip vegetables due to convenience, lack of cooking skills, taste preferences, budget constraints, and lifestyle changes.

In this study, only 19% of the respondents reported eating fruits daily. Similar findings have been reported [5]. In contrast, a Malaysian university student reported that almost half of the respondents consumed fruit at least three times per week [35]. A poor diet of fruits and vegetables is linked to several chronic diseases in adulthood [37]. This study found that the students were unaware of this health risk. The average diet of university students was high in fats. This study found that more than half of the respondents (55%) preferred junk food. While only 12% of the students consumed junk food daily, 70% frequently consumed it over a week. These findings are consistent with previous study findings [5, 35]. Students often choose fast food because it is tasty, readily available, and affordable [34].

However, Smoking and alcohol consumption were not common among the university students in this study. Bangladesh is mostly a Muslim country, and alcohol use is illegal [11]. By contrast, alcohol consumption is far more common among men and women in high‐income countries [38].

Inactivity and sedentary lifestyles are major risk factors for non‐communicable diseases [31]. This study found a high prevalence of sedentary behavior among university students. To avoid health issues linked to excessive television (TV) viewing, the American Academy of Pediatrics advises young people to watch no more than 2 h of television every day [39]. However, in this study, only 16% of the students watched TV for more than 2 h daily. The rate is low; maybe students watch alternative platforms like mobile phones, desktops, laptops, tablets, and so on. In this study, students found a higher proportion of using the internet and mobile phones daily.

Long periods of internet use have increased the daily duration of sedentary behavior among university students [31]. It is well‐recognized that excessive internet use harms mental health. It has been linked to attention‐deficit/hyperactivity disorder, sadness, loneliness, and anxiety [40]. Additional consequences of internet addiction include disturbed sleep patterns, poor personal hygiene, poor eating habits, interpersonal problems, diminished work or academic performance, headaches, eyesight problems, and psychological withdrawal symptoms [41]. In this study, a higher proportion of participants used the internet frequently. This study's findings align with Bahrain and Turkish studies [42, 43]. The proportion rate is high because the COVID‐19 pandemic drastically affects students' routines, increasing reliance on online activities for learning, socializing, and entertainment [44].

Excessive smartphone use has been linked to problems controlling emotions, impulsiveness, reduced brain function, addiction to social networking, shyness, and low self‐esteem [45]. This study found that most students used mobile phones for over 2 h daily. According to Dhaka‐based research, 57% of medical students used their phones for more than 2 h, while 34.8% used them for more than 4 h [46]. Another study reported that university students' smartphone use differed in terms of years of use, duration of daily use, daily check frequency, and daily internet use on cell phones [47]. It's essential to balance productive use and the potential drawbacks of excessive phone use.

Physical activity has numerous health advantages, particularly in preventing obesity, cardiovascular disease, diabetes, hypertension, and cancer [48]. In this study, nearly half of the students did not engage in physical activity. Conversely, in Bahrain, 82.9% practiced physical activity [31]. This difference can be due to limited outdoor space in Dhaka or people not fully understanding the importance of regular physical activity for their health.

Additionally, increasing evidence has shown that sleep affects health and eating habits [31]. Sleeping for less than 7 h daily is linked to increased food intake, poor diet quality, and weight gain [49]. The findings of this study showed that 51% of university students slept for less than 7 h per day. This may be due to poor sleep quality, spending more than an hour using social media before sleeping, or a noisy sleeping environment; Dhaka city is prone to noise pollution.

## 5 Limitations

This study has several limitations. First, self‐report measures assessed eating habits and sedentary behavior status. The collected self‐reported data may have been subject to social desirability, memory recall, and common methods biases. Second, the selected area and small sample size limited the consistency of the participants' responses. Third, we could not reach students who did not use social media. Finally, a cross‐sectional design was used, limiting the causal relationship among the variables to be interpreted in parallel.

## 6 Conclusions

The present study concludes that students in Dhaka City are at greater risk of unhealthy eating habits and a sedentary lifestyle. Furthermore, factors such as meal frequency, fruit intake, consumption of junk food, and fried food intake were associated with a greater risk of unhealthy eating behaviors. However, their physical activity levels were notably inadequate, largely characterized by a sedentary lifestyle involving substantial usage of the internet and mobile phones. Furthermore, many students did not engage in regular physical exercise. Extensive study is required among students from different parts of the country.

## 7 Recommendations

The strengths of the present findings are that they help policymakers of different educational institutes to realize that unhealthy eating habits and sedentary behavior are vital issues for young students. The study results have immediate application for healthcare professionals working with college/university students, particularly counselors in educational institutes. The findings of this study should be interpreted with caution, as they may not be fully representative of the broader Bangladeshi university student population owing to the restricted sample size. Consequently, future research endeavors require larger sample sizes to develop a nationally standardized instrument. Such efforts are vital for accurately assessing young individuals' dietary patterns and sedentary behaviors, offering valuable insights into health promotion and intervention strategies.

## Author Contributions

**Mst. Mahfuza Akter:** conceptualization, investigation, writing–original draft, methodology, validation, visualization, writing–review and editing, software, formal analysis, data curation. **Md. Jubayer Hossain:** conceptualization, investigation, writing–original draft, methodology, validation, visualization, writing–review and editing, software, formal analysis, project administration, data curation, supervision, resources.

## Conflicts of Interest

The authors declare no conflicts of interest.

## Transparency Statement

The lead author Md. Jubayer Hossain affirms that this manuscript is an honest, accurate, and transparent account of the study being reported; that no important aspects of the study have been omitted; and that any discrepancies from the study as planned (and, if relevant, registered) have been explained.

## Data Availability

The data that support the findings of this study are openly available in Eating_Habits at https://github.com/chiralbd/Eating_Habits.
